# Propionate Ameliorates *Staphylococcus aureus* Skin Infection by Attenuating Bacterial Growth

**DOI:** 10.3389/fmicb.2019.01363

**Published:** 2019-06-18

**Authors:** Soyoung Jeong, Hyun Young Kim, A Reum Kim, Cheol-Heui Yun, Seung Hyun Han

**Affiliations:** ^1^Department of Oral Microbiology and Immunology, DRI, and BK21 Plus Program, School of Dentistry, Seoul National University, Seoul, South Korea; ^2^Department of Agricultural Biotechnology and Research Institute for Agriculture and Life Sciences, Seoul National University, Seoul, South Korea

**Keywords:** *Staphylococcus aureus*, MRSA, short-chain fatty acids, propionate, D-alanine

## Abstract

*Staphylococcus aureus* causes various diseases including skin and soft tissue infections, pneumonia, gastroenteritis, and sepsis. Antibiotic-resistant *S. aureus* such as methicillin-resistant *S. aureus* (MRSA) and multidrug-resistant *S. aureus* is a serious threat in healthcare-associated settings and in the communities. In this study, we investigated the effects of short-chain fatty acids, metabolites produced by commensal bacteria, on the growth of *S. aureus* both *in vitro* and *in vivo*. Sodium propionate (NaP) most potently inhibited the growth of MRSA and multidrug-resistant clinical isolates. Of note, only NaP, but not sodium acetate (NaA) or sodium butyrate (NaB), ameliorated MRSA skin infection, significantly lowering bacterial load, excessive cytokine production, and the size and weight of abscesses approximately by twofold. In addition, interestingly, *S. aureus* deficient of lipoteichoic acids (LTA) or wall teichoic acids (WTA), which are important in bacterial physiology and antimicrobial susceptibility, was more susceptible to NaP than the wild-type. Furthermore, *S. aureus* deficient of D-alanine motifs common in LTA and WTA was more susceptible to NaP, its growth being almost completely inhibited. Concordantly, MRSA treated with an inhibitor of D-alanylation on LTA and WTA was more susceptible to NaP, and co-treatment of NaP and a D-alanylation inhibitor further decreased the pathology of MRSA skin infection. Collectively, these results demonstrate that NaP ameliorates MRSA skin infection by attenuating the growth of *S. aureus*, and suggest an alternative combination treatment strategy against *S. aureus* infection.

## Introduction

*Staphylococcus aureus*, which frequently colonizes humans, is a major pathogen that causes various diseases including skin and soft tissue infections, pneumonia, and gastroenteritis, and the most frequent bacterium associated with sepsis (Lowy, 1998; Alberti et al., 2002). Skin and soft tissue infections represent approximately 90% of *S. aureus* infections and can lead to the spread of *S. aureus* to other parts of the body, often resulting in serious diseases such as bacteremia or pneumonia (DeLeo et al., 2010). Of note, *S. aureus* is especially adept at acquiring antibiotic resistance. For instance, methicillin-resistant *S. aureus* (MRSA) is a serious threat that has become more prevalent. In the US, MRSA is predicted to kill approximately 19,000 patients per year, which is similar to the number of deaths by AIDS, tuberculosis, and hepatitis combined (Boucher and Corey, 2008). MRSA now causes not only healthcare-associated, but also community-associated infections (Klevens et al., 2007; DeLeo et al., 2010). The emergence of vancomycin-resistant *S. aureus* and multidrug-resistant *S. aureus* is a growing challenge (Walters et al., 2015). In addition, resistance to new classes of antibiotics (Ventola, 2015) is emphasizing the limited treatment options. Furthermore, since there is no vaccine available to prevent *S. aureus* infection (Giersing et al., 2016), a novel strategy to combat antibiotic-resistant infection is needed.

Short-chain fatty acids (SCFAs) are metabolites produced by commensal bacteria when dietary fibers and non-digestible carbohydrates are fermented in the colon (Topping and Clifton, 2001). The main SCFAs in humans are acetate, propionate, and butyrate, which are produced in a molar ratio of approximately 60:20:20 (Cummings, 1981; Topping and Clifton, 2001). The concentration of SCFAs is in the range of 70–140 mM where they are the most abundant, and SCFAs are also found in the blood in micromolar concentrations (Cummings et al., 1987). SCFAs have various roles in the host, simply acting as energy sources for colonocytes, and leading to enhanced mucus and antimicrobial peptides production (Makki et al., 2018). A high fiber diet, which results in an increased production of SCFAs, promotes colon integrity and protects against allergy and inflammatory conditions (Hou et al., 2011; Tan et al., 2016). In addition, SCFAs regulate the immune system to maintain host immune homeostasis primarily in the gut, by inducing regulatory T cell development (Smith et al., 2013; Koh et al., 2016).

Apart from their regulatory roles in the host, SCFAs have various antimicrobial effects on some pathogenic bacteria (Sun and O’Riordan, 2013). The degree of toxicity mediated by SCFAs varies among different bacterial species. Butyrate inhibits the growth of *Helicobacter pylori* by exhibiting destructive effects on its cell envelope (Yonezawa et al., 2012). Similarly, acetic acid inhibits the growth of *Escherichia coli* (Roe et al., 2002). In addition, butyrate regulates the virulence of *Salmonella enterica* serovar Typhimurium and Enteritidis by downregulating *Salmonella* pathogenicity island 1 gene expression (Gantois et al., 2006), while propionate suppresses *S. enterica* serovar Typhimurium invasion (Hung et al., 2013). Furthermore, it has been suggested that the fermentation products of *Propionibacterium acnes*, which contain various metabolites and proteins including propionic acid and butyric acid, inhibit *S. aureus* colonization (Shu et al., 2013). Moreover, propionic acid has been suggested to inhibit *S. aureus* growth by reducing bacterial intracellular pH (Wang et al., 2014). However, although propionic acid can change the pH of the medium and the *in vivo* environment, the effects of the three SCFAs, sodium acetate (NaA), sodium propionate (NaP), or sodium butyrate (NaB), which do not affect pH, have not been studied. In this study, the effects of SCFAs on *S. aureus in vitro* and *in vivo* were studied and an alternative combination treatment strategy to control antibiotic-resistant *S. aureus* infections was investigated.

## Materials and Methods

### Reagents and Chemicals

SCFAs were purchased from Sigma-Aldrich Inc. (St. Louis, MO, United States). SCFAs were dissolved in endotoxin-free distilled water (Dai Han Pharm Co. Ltd., Seoul, South Korea) and filtered with a syringe filter (0.2 μm) purchased from Corning (Corning, NY, United States) prior to use. Luria-Bertani (LB) broth was purchased from LPS solution (Daejeon, South Korea). Trypticase soy broth (TSB) and Bacto agar were purchased from BD Biosciences (Franklin Lakes, NJ, United States). 2,2,2-Tribromoethanol and 2-methyl-2-butanol were purchased from Sigma-Aldrich Inc. Amsacrine (AMSA) was purchased from Abcam (Cambridge, United Kingdom). Hematoxylin and eosin were purchased from Sigma-Aldrich Inc. and BBC Biochemical (Mount Vernon, WA, United States), respectively. Crystal violet and safranin were purchased from Sigma-Aldrich Inc. Iodide solution was purchased from Samchun Chemicals (Seoul, South Korea).

### Bacterial Strains and Culture Conditions

Bacterial strains used in this study are listed in Table 1. MRSA USA300 was obtained from the Nebraska Transposon Mutant Library (Omaha, NE, United States). Clinically isolated *S. aureus* strains were obtained from the National Culture Collection for Pathogens (Osong, South Korea). Strains and isolation sites, in parentheses, are as follows: MRSA NCCP 11485 (urine), 11486 (urine), 14565 (blood), 14566 (abscess), 14567 (abscess), 14568 (catheter tip), 14569 (abscess), 14748 (nose), 14750 (blood), 14751 (ear), 14769 (transtracheal aspirates), and vancomycin intermediate-resistant *S. aureus* (VISA) NCCP 13846 (pus), 13853 (pus), and 13863 (pus). These strains were cultured in TSB at 37°C with shaking. Wild-type (WT) *S. aureus* RN4220 (Novick, 1991) and its lipoteichoic acid (LTA)-deficient (Δ*ltaS*) (Oku et al., 2009), wall teichoic acid (WTA)-deficient (Δ*tagO*) (Kaito and Sekimizu, 2007), lipoprotein (LPP)-deficient (Δ*lgt*) (Kurokawa et al., 2009), D-alanine-deficient (Δ*dltA*) (Kaito and Sekimizu, 2007) mutants, and their complement strains of each (Δ*ltaS*/pM101-*ltaS*) (Oku et al., 2009), Δ*tagO*/pS*tagO* (Park et al., 2010), Δ*lgt*/pS*lgt* (Kurokawa et al., 2009), and Δ*dltA*/p0793 (Kaito and Sekimizu, 2007) were kindly provided by Prof. Bok-Luel Lee (Pusan National University, Busan, South Korea). WT, Δ*lgt*, Δ*lgt*/pS*lgt*, Δ*dltA*, and Δ*dltA*/p0793 *S. aureus* were cultured in LB broth at 37°C with shaking (Kaito and Sekimizu, 2007; Kurokawa et al., 2009). Δ*ltaS*, Δ*ltaS*/pM101-*ltaS*, Δ*tagO*, and Δ*tagO*/pS*tagO S. aureus* were cultured in LB broth at 30°C with shaking as previously described (Kaito and Sekimizu, 2007; Oku et al., 2009).

**Table 1:** Bacterial strains used in this study.

Strain	Characteristics	Source or references
USA300	MRSA	Nebraska Transposon Mutant Library
RN4220 WT	Lab strain	Novick, 1991
RN4220 Δ*ltaS*	Loss of LTA	Oku et al., 2009
RN4220 Δ*tagO*	Loss of WTA	Kaito and Sekimizu, 2007
RN4220 Δ*lgt*	Loss of lipoprotein lipid modification	Kurokawa et al., 2009
RN4220 Δ*dltA*	Loss of D-ala modification in LTA and WTA	Kaito and Sekimizu, 2007
RN4220 Δ*ltaS*/pM101-*ltaS*	Δ*ltaS* strain containing a plasmid harboring the *ltaS* gene	Oku et al., 2009
RN4220 Δ*tagO*/pS*tagO*	Δ*tagO* strain containing a pS*tagO* plamid	Park et al., 2010
RN4220 Δ*lgt*/pS*lgt*	Δ*lgt* strain containing a plasmid harboring the *lgt* gene	Kurokawa et al., 2009
RN4220 Δ*dltA*/p0793	Δ*dltA* strain containing a plasmid harboring intact *dltABCD*	Kaito and Sekimizu, 2007
NCCP 11485	Urine; resistance to methicillin, oxacillin, penicillin, erythromycin, cefazolin, amoxicillin/clavulanic acid, gentamicin, tetracycline, mupirocin	National Culture Collection for Pathogens
NCCP 11486	Urine; resistance to methicillin, oxacillin, penicillin, erythromycin, clindamycin, cefazolin, amoxicillin/clavulanic acid, ofloxacin, tetracycline, rifampin	National Culture Collection for Pathogens
NCCP 14565	Blood; resistance to methicillin, penicillin, tetracycline, linezolid, quinupristin/dalfopristin	National Culture Collection for Pathogens
NCCP 14566	Abscess; resistance to methicillin, penicillin, cefoxitin	National Culture Collection for Pathogens
NCCP 14567	Abscess, resistance to methicillin, penicillin, cefoxitin, erythromycin, clindamycin, tetracycline	National Culture Collection for Pathogens
NCCP 14568	Catheter tip; resistance to methicillin, penicillin, cefoxitin, ofloxacin, erythromycin, clindamycin, tetracycline	National Culture Collection for Pathogens
NCCP 14569	Abscess; resistance to methicillin, penicillin, cefoxitin, erythromycin	National Culture Collection for Pathogens
NCCP 14748	Nose; resistance to methicillin, penicillin, cefoxitin, erythromycin	National Culture Collection for Pathogens
NCCP 14750	Blood; resistance to methicillin, penicillin, cefoxitin, erythromycin	National Culture Collection for Pathogens
NCCP 14751	Ear; resistance to methicillin, penicillin, cefoxitin, ofloxacin, erythromycin, clindamycin, tetracycline	National Culture Collection for Pathogens
NCCP 14769	Transtracheal aspirates; resistance to methicillin	National Culture Collection for Pathogens
NCCP 13846	Pus; intermediate resistance to vancomycin, resistance to methicillin, oxacillin, penicillin, erythromycin	National Culture Collection for Pathogens
NCCP 13853	Pus; intermediate resistance to vancomycin, resistance to methicillin, oxacillin, penicillin, erythromycin, clindamycin, amoxicillin/clavulanic acid, ofloxacin, gentamicin, tetracycline	National Culture Collection for Pathogens
NCCP 13863	Pus; intermediate resistance to vancomycin, resistance to methicillin, oxacillin, penicillin, erythromycin, amoxicillin/clavulanic acid, gentamicin, tetracycline	National Culture Collection for Pathogens

### Effects of SCFAs or AMSA on the Growth of *S. aureus in vitro*

A single colony was inoculated and cultured overnight. One percent of an overnight culture was inoculated to fresh medium (LB or TSB accordingly) in the presence or absence of various doses of SCFAs (1.56, 3.13, 6.25, 12.5, 25, 50, or 100 mM) and/or AMSA (1.56, 3.13, 6.25, 10, 12.5, 20, or 25 μg/ml) in flat bottom, non-coated polystyrene 96-well plates (Thermo Scientific, Waltham, MA, United States). Bacteria were cultured at 30 or 37°C accordingly with shaking and optical density at 600 nm was measured hourly using a spectrophotometer (Molecular Devices, Sunnyvale, CA, United States). Growth studies were also conducted in 50 ml conical tubes, in a flask to volume ratio of 10:1, showing the same result (data not shown).

### Minimum Inhibitory Concentration/Minimum Bactericidal Concentration (MIC/MBC) Test

The MIC/MBC test was conducted using the microdilution method adopted from the Clinical and Laboratory Standards Institute (CLSI) guidelines (CLSI, 2012). Bacteria at 5 × 10^5^ CFU/ml were inoculated in media containing serially diluted antimicrobial substances (0, 3.9, 7.8, 15.6, 31.3, 62.5, 125, 250, or 500 mM SCFAs, with 10 μg/ml AMSA where indicated) and cultured for 24 h. The MIC was defined as the minimum concentration that resulted in no visible growth after 24 h. Optical density at 600 nm was measured to confirm no growth. To determine the MBC, wells that did not result in bacterial growth were inoculated in fresh media, free of antimicrobial substances. Optical density at 600 nm was measured after 24 h.

### Murine Skin Infection

Animal experiments were conducted under the approval of Institutional Animal Care and Use Committee of Seoul National University (SNU-170518-5 and SNU-181002-2). Eight- to ten-week-old female C57BL/6 mice purchased from Orient Bio (Seongnam, South Korea) were used for experiments. *S. aureus* skin infection model was used with slight modification (Malachowa et al., 2013). Briefly, MRSA cultured to mid-log phase was washed and resuspended in endotoxin-free distilled water alone, or in endotoxin-free distilled water containing 50 mM SCFA and/or 10 μg/ml AMSA, to a final concentration of 3 × 10^7^ CFU/ml. Control samples containing SCFA and/or AMSA alone were also prepared. Mice were anesthetized with a mixture of 2,2,2-tribromoethanol and 2-methyl-2-butanol. The flank area of mice was shaved with an electric hair clipper and depilatory cream. After disinfecting the injection sites with ethanol, mice were challenged with 3 × 10^6^ CFU MRSA in 100 μl endotoxin-free distilled water. Animals were monitored daily for three days. On day 3, after euthanasia, abscess length and width were measured with a digital caliper (Mitutoyo Corporation, Kawasaki, Japan) to obtain abscess size. Abscesses were excised and weighed. Abscesses were homogenized, serially diluted, and plated on TSB agar to measure bacterial burden.

### Enzyme-Linked Immunosorbent Assay (ELISA)

Homogenates of abscesses were centrifuged twice and the supernatants were stored at -80°C until use. The levels of IL-1β and IL-6 in the supernatants were measured using commercial ELISA kits (Biolegend, San Diego, CA, United States).

### Histological Analysis

Three days after subcutaneous infection, skin abscesses were excised and cryosectioned longitudinally onto slide glasses at 10 μm using a cryocut microtome 1860 (Leica, Wetzlar, Germany). The sections were analyzed by hematoxylin and eosin (H&E) staining as previously described (Nurul et al., 2018) or Gram-staining.

### Statistical Analysis

All *in vitro* experiments were conducted at least three times. For each experiment, the mean values ± standard deviation (SD) were obtained from triplicate samples. For *in vivo* studies, data are represented as mean values ± standard error of mean (SEM). Data were analyzed with GraphPad Prism software. Statistical significance was measured using the paired student’s *t*-test to compare between the groups. Asterisks indicate statistically significant differences; ^∗^*P* < 0.05, ^∗∗^*P* < 0.01, ^∗∗∗^*P* < 0.001, ^∗∗∗∗^*P* < 0.0001.

## Results

### SCFAs Inhibit the Growth of MRSA

**Figure 1:**
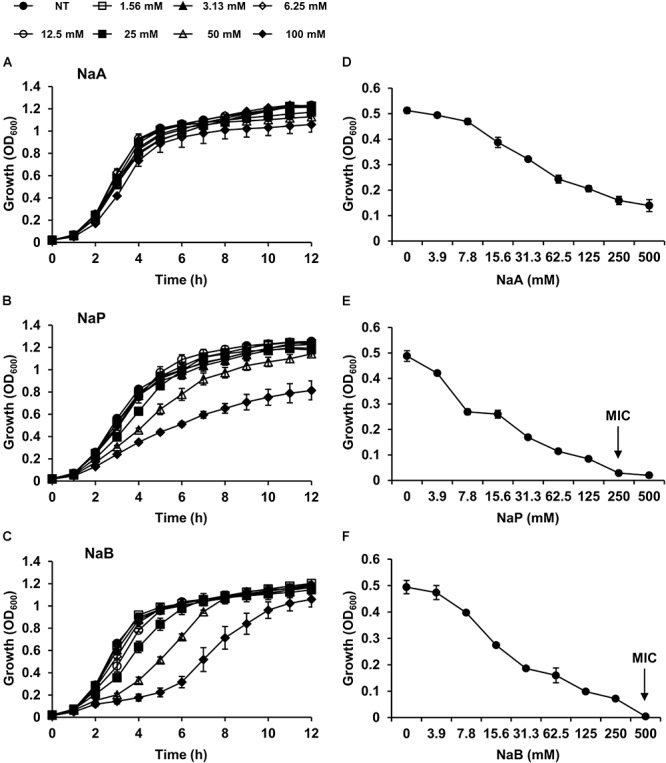
SCFAs dose-dependently attenuate the growth of MRSA. MRSA was cultured either in the presence or absence of different doses of **(A)** NaA, **(B)** NaP, or **(C)** NaB. Optical density at 600 nm was measured hourly. **(D,E)** The MIC/MBC test was conducted using the microdilution method with **(D)** NaA, **(E)** NaP, or **(F)** NaB. The MIC, the concentration of NaP and NaB which completely inhibited growth, is indicated with an arrow. Data shown are the mean values ± SD of triplicate samples and are representative of at least three similar independent experiments.

To determine the inhibitory effects of SCFAs, MRSA was cultured in the presence or absence of different doses of NaA, NaP, or NaB, and the growth of MRSA was examined. All three SCFAs inhibited the growth of MRSA in a dose-dependent manner. NaP and NaB potently inhibited the growth of MRSA, while NaA had a minimal impact (Figure 1A–C). Interestingly, at 100 mM, the inhibitory property of NaP was maintained until 12 h after the treatment, whereas the inhibitory effect of NaB did not (Figure 1B,C). NaB prolonged the lag phase of MRSA. Next, to investigate if the effects of SCFAs are bacteriostatic or bactericidal, the MIC and MBC were determined. The MICs of NaP and NaB were 250 and 500 mM, respectively, while NaA did not have an MIC in the concentration range tested (Figure 1D,E). There was no MBC for all three SCFAs at the concentrations tested, indicating that SCFAs have bacteriostatic, rather than bactericidal, effects. To confirm that NaP had a bacteriostatic effect, the MIC/MBC test was conducted with higher concentrations of NaP. There was no MBC even at 3 M NaP (Supplementary Figure S1). Since agents are considered bacteriostatic when the MBC is greater than four times the MIC (French, 2006), NaP had a bacteriostatic effect. In addition, NaP did not affect the pH of the extracellular medium throughout the experiment (data not shown). Growth studies and the MIC/MBC test demonstrated that NaP was the most potent in inhibiting MRSA growth. In addition, when the morphology of *S. aureus* in the presence or absence of NaP was analyzed with scanning electron microscopy, *S. aureus* treated with NaP showed no morphological differences compared to non-treated (NT) *S. aureus* (Supplementary Figure S2). To extend our observations, different strains of *S. aureus* were cultured in the presence or absence of NaP or NaB. NaP and NaB inhibited the growth of various clinically isolated MRSA and VISA strains isolated from different sites, including multidrug-resistant *S. aureus* (Table 2). Similar to the results in Figure 1, NaB prolonged the lag phase of MRSA. The inhibitory effect of NaP was maintained until stationary phase for most strains, whereas that of NaB was not. These results suggest that NaP has a potent inhibitory effect on MRSA growth.

**Table 2:** The effects of NaP and NaB on the growth of clinically isolated, antibiotic-resistant *S. aureus*.

Strain (NCCP)	Mid-log phase^a^			Stationary phase^a^		
	NT	50 mM NaP	50 mM NaB	NT	50 mM NaP	50 mM NaB
**Methicillin-resistant *S. aureus* (MRSA)**		
11485^†^	0.456	0.222	0.105	1.010	0.700	0.427
11486^†^	0.462	0.221	0.164	0.950	1.001^b^	1.022^b^
14565^†^	0.844	0.423	0.265	1.061	0.822	1.027^b^
14566^†^	0.755	0.379	0.184	0.930	0.805	0.867
14567^†^	0.444	0.192	0.111	0.860	0.485	0.693
14568^†^	0.438	0.220	0.180	0.892	0.947^b^	0.920^b^
14569^†^	0.763	0.477	0.269	0.966	0.937^b^	1.044^b^
14748^†^	0.594	0.283	0.351	1.037	0.610	1.017^b^
14750^†^	0.805	0.428	0.275	0.995	0.900	1.026^b^
14751^†^	0.447	0.245	0.194	0.901	0.874	0.896^b^
14769	0.489	0.290	0.199	0.898	0.887^b^	0.904^b^
**Vancomycin intermediate-resistant *S. aureus* (VISA)**		
13846^†^	0.498	0.247	0.108	1.010	0.727	0.556
13853^†^	0.684	0.375	0.645^b^	0.999	0.848	1.047^b^
13863^†^	0.674	0.384	0.343	1.089	0.691	0.949

^a^*OD_*600*_ of the same culture; ^*b*^not significant compared to NT (*P* > 0.05). ^†^Multidrug-resistant*.

### NaP Alleviates the Pathology of MRSA in Murine Skin Infection

**Figure 2:**
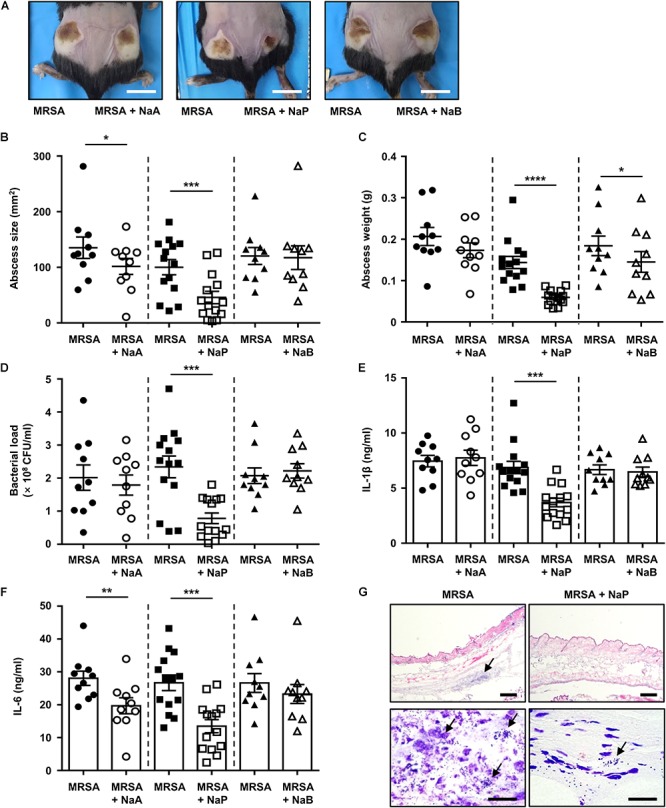
NaP reduces bacterial load and dermonecrosis in murine MRSA skin infection. C57BL/6 mice were subcutaneously infected with 3 × 10^6^ CFU MRSA USA300 alone (left), or together with 50 mM NaA, NaP, or NaB (right) (*n* = 10–14 per group). **(A)** Images of abscesses on day 3. Scale bars indicate 1 cm. On day 3, after euthanasia of mice, **(B)** size and **(C)** weight of abscesses were measured. **(D)** Bacterial load was measured by excising and homogenizing abscesses aseptically, and spotting homogenates on TSB agar plates. Homogenates were centrifuged and the supernatants were used to measure **(E)** IL-1β and **(F)** IL-6. **(G)** Abscesses were cryosectioned and evaluated for histopathology by H&E staining and Gram staining. Scale bars indicate 200 and 20 μm for top and bottom panels, respectively. Data are represented as mean values ± SEM, and statistical significance was measured with the student’s *t*-test. ^∗^*P* < 0.05, ^∗∗^*P* < 0.01, ^∗∗∗^*P* < 0.001, ^∗∗∗∗^*P* < 0.0001.

As MRSA is a major cause of skin and soft tissue infections, the effects of SCFAs on MRSA skin infection *in vivo* were investigated by using a murine skin infection model (Malachowa et al., 2013). Mice were subcutaneously infected with MRSA with or without NaA, NaP, or NaB. The results showed that abscesses formed by day 3 post-infection (Figure 2A). The abscess size and weight were significantly decreased upon NaP co-injection with MRSA, while NaA or NaB co-injection did not greatly affect them (Figure 2B,C). When the abscesses were excised and homogenized to determine bacterial load, NaP, but not NaA or NaB, resulted in a significantly lower bacterial load, showing a twofold reduction (Figure 2D). Mice co-injected with NaP had a significantly lower production of IL-1β, a signature cytokine of *S. aureus* abscess formation (Cho et al., 2012), compared with those injected with NaA or NaB (Figure 2E). IL-6 levels were also significantly lower for NaP-injected mice (Figure 2F). Interestingly, NaB treatment did not affect lesion size, bacterial load, or cytokine expression though it had some inhibitory effects on the growth of MRSA *in vitro*. Histological analysis of abscesses demonstrated higher immune cell infiltration and bacterial clusters, both indicated with an arrow, in mice infected with MRSA (Figure 2G, left). In NaP-injected abscesses, there was less immune cell infiltration, and a lower number of bacterial clusters was observed compared with NT abscesses (Figure 2G, right). NaP alone, at the concentration used in the skin infection study, was not toxic to mice and did not result in pathology or cytokine expression (data not shown). These results indicate that NaP could reduce the pathology of MRSA skin infection, by lowering bacterial load, excessive cytokine release, and size and weight of abscesses.

### *S. aureus* With D-Alanine-Deficient LTA and WTA Is More Susceptible to the Growth Inhibition by NaP

**Figure 3:**
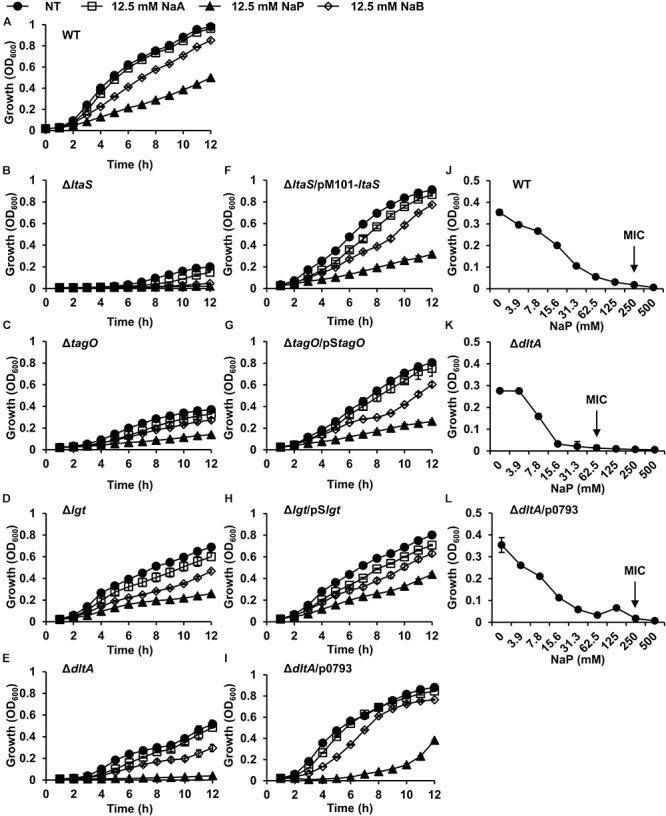
*S. aureus* with D-alanine-deficient LTA and WTA is susceptible to the growth inhibition by NaP. **(A)** WT, **(B)** Δ*ltaS*, **(C)** Δ*tagO*, **(D)** Δ*lgt*, **(E)** Δ*dltA*, **(F)** Δ*ltaS*/pM101-*ltaS*, **(G)** Δ*tagO*/pS*tagO*, **(H)** Δ*lgt*/pS*lgt* or **(I)** Δ*dltA*/p0793 was inoculated and cultured in the presence or absence of 12.5 mM of NaA, NaP, or NaB. The optical density at 600 nm was measured hourly. **(J–L)** The MIC/MBC test for NaP was conducted using the microdilution method with **(J)** WT, **(K)** Δ*dltA*, or **(L)** Δ*dltA*/p0793. The MIC, the concentration of NaP which completely inhibited growth, is indicated with an arrow. Data shown are the mean values ± SD of triplicate samples and are representative of at least three similar independent experiments.

LTA, WTA, and LPP are cell wall components of Gram-positive bacteria that play important roles in bacterial growth, division, and antimicrobial susceptibility (Schmaler et al., 2010; Brown et al., 2013; Percy and Grundling, 2014). To gain insight into the action mechanism of the growth inhibition by NaP, we compared the effects of SCFAs on the growth of WT *S. aureus* to that of LTA-deficient (Δ*ltaS*), WTA-deficient (Δ*tagO*), or LPP-deficient (Δ*lgt*) *S. aureus*. The growth of the parent strain *S. aureus* RN4220 was inhibited by SCFAs in a dose-dependent manner (Supplementary Figure S3). Compared to the WT (Figure 3A), LTA-deficient or WTA-deficient *S. aureus* was more susceptible to the growth inhibition by SCFAs, especially NaP (Figure 3B,C). However, LPP-deficient *S. aureus* exhibited a similar growth pattern when cultured in the presence of different SCFAs (Figure 3D). Since LTA and WTA share D-alanine motifs in common (Weidenmaier and Peschel, 2008; Reichmann et al., 2013), we next examined the effects of SCFAs on the growth of *S. aureus* which lacks D-alanine motifs on LTA and WTA (Δ*dltA*) (Kaito and Sekimizu, 2007). As shown in Figure 3E, *S. aureus* with D-alanine-deficient LTA and WTA was substantially more susceptible to NaP than the WT. Complement strains of each mutant *S. aureus* had similar growth patterns as the WT (Figure 3F–I). Moreover, the MIC of NaP was 62.5 mM for *S. aureus* with D-alanine-deficient LTA and WTA, a value fourfold lower than that for the WT (Figure 3J,K). As expected, *S. aureus* with D-alanine-deficient LTA and WTA complemented with intact *dltABCD* had an equal MIC value as the WT (Figure 3L). Therefore, D-alanine motifs on LTA and WTA are important in modulating the susceptibility to NaP.

### D-Alanylation Inhibition Increases Susceptibility of MRSA to the Growth Inhibition by NaP

**Figure 4:**
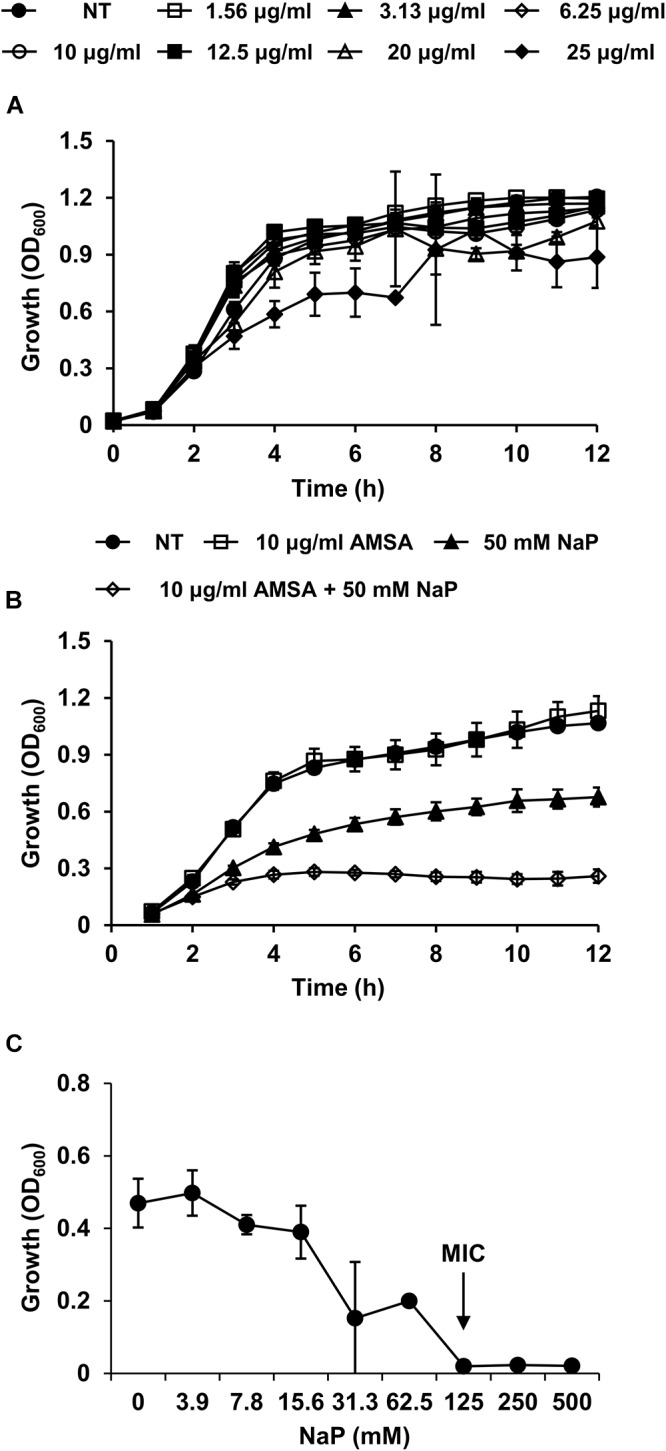
D-Alanylation inhibition increases the susceptibility of MRSA to the growth inhibition by NaP. **(A)** MRSA was cultured in the presence of different doses of AMSA, a D-alanylation inhibitor. Optical density was measured hourly. **(B)** MRSA USA300 was cultured in the presence of 10 μg/ml AMSA, 50 mM NaP, or both. Optical density was measured hourly. **(C)** The MIC/MBC test was conducted using the microdilution method with NaP in the presence of 10 μg/ml AMSA. The MIC, the concentration of NaP which completely inhibited growth, is indicated with an arrow. Data shown are the mean values ± SD of triplicate samples and are representative of at least three independent experiments.

We next sought to verify the importance of D-alanine residues in the growth inhibition by NaP. A D-alanylation inhibitor, AMSA, which inhibits DltB, the transmembrane protein that is essential for D-alanylation of teichoic acids (Pasquina et al., 2016), was used to study the role of D-alanine residues on LTA and WTA in MRSA. When MRSA was cultured in the presence of different doses of AMSA alone, the growth of MRSA was largely unaffected up to 25 μg/ml (Figure 4A). To note, the MIC of AMSA was 100 μg/ml (Supplementary Figure S4). AMSA was used at 10 μg/ml, a concentration that inhibited D-alanylation but had minimal effects on bacterial growth, to determine its effect when used together with NaP. Co-treatment of 10 μg/ml AMSA and 50 mM NaP resulted in ca. 80% inhibition of MRSA growth (Figure 4B). Moreover, the MIC of NaP was reduced twofold, to 125 mM, when AMSA was co-treated (Figure 4C). When the fractional inhibitory concentration index (FICI) (Tascini et al., 2000) was calculated, NaP and AMSA had partial synergy (FICI = 0.6). These results demonstrate that D-alanine residues on LTA and WTA are important in modulating the susceptibility of MRSA to NaP.

### Co-treatment of NaP and a D-Alanylation Inhibitor Further Ameliorates MRSA Skin Infection

**Figure 5:**
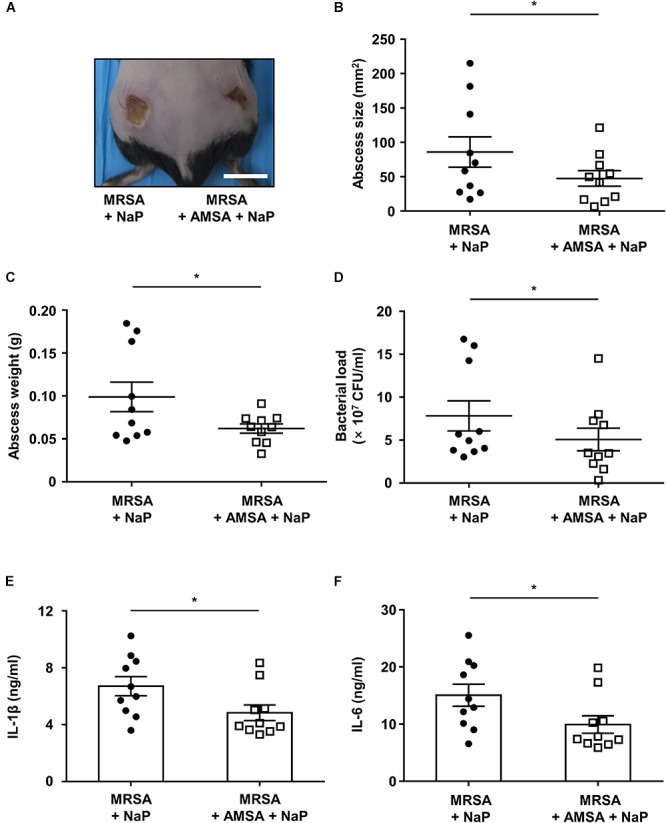
NaP reduces bacterial load in murine skin infection when co-treated with a D-alanylation inhibitor. C57BL/6 mice were subcutaneously infected with 3 × 10^6^ CFU MRSA USA300 together with 50 mM NaP (left), or with 50 mM NaP and 10 μg/ml AMSA (right) (*n* = 10). **(A)** Images of abscesses on day 3. Scale bar indicates 1 cm. On day 3, after euthanasia, **(B)** size, and **(C)** weight of abscesses were measured. **(D)** Bacterial load was measured by excising and homogenizing abscesses aseptically, and spotting homogenates on TSB agar plates. Homogenates were centrifuged and the supernatants were used to measure **(E)** IL-1β and **(F)** IL-6. Data are represented as mean values ± SEM, and statistical significance was measured with the student’s *t*-test. ^∗^*P* < 0.05.

Since the co-treatment of AMSA and NaP led to almost complete inhibition of MRSA *in vitro*, we next studied their effects *in vivo*. Mice were subcutaneously infected with MRSA together with NaP alone or AMSA and NaP (Figure 5A). To distinguish the effects of AMSA alone, mice infected with MRSA and AMSA were compared with those infected with MRSA alone. Although AMSA alone slightly decreased the abscess size, it did not affect the bacterial load, cytokine expression, or abscess weight (Supplementary Figure S5). Interestingly however, co-injection of AMSA and NaP further reduced size and weight of the abscess compared to NaP injection alone (Figure 5B,C). Moreover, a combination of AMSA and NaP further reduced the number of bacteria recovered from abscesses, when compared with NaP alone (Figure 5D). The expression of pro-inflammatory cytokines, IL-1β and IL-6, was also further reduced (Figure 5E,F). Collectively, these results indicate that NaP inhibits MRSA infection both *in vitro* and *in vivo*, suggesting combination treatment of AMSA and NaP as an efficient treatment strategy to control antibiotic-resistant *S. aureus* infections.

## Discussion

Due to the increased prevalence in infections caused by multidrug-resistant *S. aureus* concordant with a limited availability of antibiotics, alternative therapeutic strategies against *S. aureus* infections are urgently needed. Recently, combination therapy and the concept of synthetic lethality have been gaining attention as a way to overcome antibiotic-resistant *S. aureus* infection (Campbell et al., 2011; Dilworth et al., 2014). Combination therapy has been thought to be more effective, and less prone to resistance. In this study, we demonstrated that NaP inhibits the growth of MRSA, and ameliorates *S. aureus* skin infection. A combination of NaP and AMSA (a D-alanylation inhibitor) more potently inhibited MRSA infection, suggesting combination treatment as an efficient strategy to control multidrug-resistant *S. aureus* infections.

In this study, we have used SCFAs for the purpose of alleviating the symptoms of MRSA infections, and shown that NaP potently inhibits the growth of MRSA both *in vitro* and *in vivo*. The inhibitory effect of NaP seems to be a general phenomenon among various *S. aureus* strains, since the growth of USA300 and clinically isolated antibiotic-resistant *S. aureus* including MRSA and VISA was inhibited. Similarly, it has been previously suggested that the fermentation products of *P. acnes*, which contain butyric acid, 3-hydroxy-butyric acid, lactic acid, propionic acid, and ethanol, can interfere with *S. aureus* colonization in a wound model (Shu et al., 2013). Although the involvement of other proteins and/or metabolites in the fermentation products of *P. acnes* cannot be excluded, propionic acid seems to be responsible for the interference. Propionic acid, of pH 3.5, inhibited *S. aureus* colonization in the wound (Shu et al., 2013). Furthermore, another study has reported the growth inhibitory properties of propionic acid, whose effect is more potent at low pH than high pH (Wang et al., 2014). As a high concentration of propionic acid would result in an acidic environment, propionic acid could lead to side effects such as skin irritation and corrosion (Basketter et al., 2012). On the other hand, in this study, we have shown that 50 mM NaP, which is not acidic and does not change the extracellular pH, ameliorates MRSA skin infection.

As NaP has a bacteriostatic effect on *S. aureus*, it is likely that NaP interferes with bacterial metabolism. However, the exact mechanism by which NaP inhibits *S. aureus* growth is unclear. There are a few possible explanations. First, it has been suggested that bacteriostatic agents can change the metabolic state of the bacterium, attenuating cellular respiration (Lin et al., 2014; Lobritz et al., 2015). Therefore, NaP, which also has a bacteriostatic effect, may interfere with metabolic pathways important for bacterial growth, such as cellular respiratory pathways including glycolysis, tricarboxylic acid cycle, or oxidative phosphorylation. Second, when the effect of NaP on the growth of MRSA deficient of tricarboxylic acid cycle enzymes was investigated, the growth of these mutants was inhibited to a higher degree by NaP (data not shown), suggesting that bacterial metabolism may be involved in the growth inhibition by NaP. Third, propionate may interfere with pyruvate decarboxylation, which is true for *Rhodopseudomonas sphaeroides* (Maruyama and Kitamura, 1985), where propionate was converted to propionyl-CoA, and then interfered with the pyruvate dehydrogenase complex. The growth inhibition by propionyl-CoA was dependent on coenzyme A concentration, as it was competitive with respect to coenzyme A. Since *S. aureus* also has a putative propionate CoA-transferase (Selmer et al., 2002), NaP may be converted to propionyl-CoA and interfere with the pyruvate decarboxylation in *S. aureus*. Moreover, RNA sequencing analysis showed substantial metabolic changes in NaP-treated *S. aureus* (data not shown). Even though further studies are needed to address these possible mechanisms, NaP seemingly, at least in part, affects the metabolic pathways of *S. aureus*.

We demonstrated that *S. aureus* is more susceptible to the growth inhibition by NaP when D-alanine motifs on LTA and WTA are absent, by using AMSA, a D-alanylation inhibitor, or *S. aureus* with D-alanine-deficient LTA and WTA. Since teichoic acids play important roles in bacterial physiology, resistance to antimicrobials, and pathogenesis, teichoic acid biosynthesis has been considered to be potential targets for controlling and combination therapy of *S. aureus* infections (Farha et al., 2013; Pasquina et al., 2013; Wang et al., 2013). Especially, D-alanine residues on LTA and WTA are important for many physiological processes including regulation of autolytic enzymes, colonization, and virulence (Fischer et al., 1981; Collins et al., 2002; Weidenmaier et al., 2004). Moreover, the absence of D-alanylation leads to increased susceptibility of bacteria to antimicrobial peptides, neutrophil killing, and antibiotics (Peschel et al., 1999; Peschel et al., 2000; Collins et al., 2002). It has been reported that D-alanylation of LTA and WTA increases the cell wall density, and D-alanine residues confer resistance to antimicrobial peptides by decreasing permeability (Saar-Dover et al., 2012). It is probable that when D-alanine residues on LTA and WTA are absent, NaP may be able to diffuse more easily into *S. aureus* to interfere with bacterial physiology. Furthermore, the varying degree of susceptibility among the different strains of *S. aureus* may reflect differences in the level of D-alanylation of teichoic acids, considering that increased D-alanylation has been reported for antibiotic-resistant *S. aureus* clinical isolates (Bertsche et al., 2013). Although further studies are needed to elucidate the connection between NaP susceptibility and D-alanylation, we believe that there is a correlation and suggest that combination therapy of NaP with a D-alanylation inhibitor may be an effective strategy to control multidrug-resistant *S. aureus* infections.

NaP significantly decreased the pathology of MRSA skin infection, by lowering the bacterial load, excessive cytokine expression, and abscess formation. SCFAs are well-known to have immunomodulatory effects (Koh et al., 2016). In fact, NaP has been reported to inhibit innate immune cell responses to microbial stimulation (Ciarlo et al., 2016). However, in this study, the decrease in MRSA skin infection is likely due to the decreased absolute number of bacteria and the growth inhibitory properties of NaP, as a bacteriostatic agent also had antibacterial effects in *S. aureus* lung infection (Jacqueline et al., 2014). One may think that NaP affected the host immune response. Although the immunomodulatory effect of NaP cannot be excluded, it is likely that the effect of NaP on the host immune system is minor. First, although NaB more potently regulates the immune system than NaP by inhibiting histone deacetylation (Chang et al., 2014; Park et al., 2019), NaB did not decrease MRSA pathology in our skin infection model. Second, cytokine expression decreased only when the bacterial load was lower. This suggests that cytokine expression was decreased because of a reduction in the absolute number of bacteria. Third, when AMSA was co-treated with NaP, bacterial load, cytokine expression, and abscess formation were further reduced. Since targeting bacteria further ameliorated MRSA skin infection, the decreased pathology of MRSA seems to be a result of decreased bacterial load. Therefore, NaP ameliorates MRSA skin infection by attenuating bacterial growth, and the effect of NaP on the host is minimal.

NaP inhibited the growth of all strains of *S. aureus* tested, including clinically isolated multidrug-resistant *S. aureus*, and decreased the pathology of MRSA *in vivo*. Since propionate is a metabolite present in our body, it is biocompatible, and is likely to have fewer or no side effects in the host compared with other antibiotics. Indeed, treatment with NaP alone did not result in pathology or inflammation (data not shown). Interventions that increase propionate production in the host (Chambers et al., 2015) may be applied for *S. aureus* infections. In addition, co-treatment of NaP and a D-alanylation inhibitor almost completely inhibited *in vitro* growth of MRSA and further reduced dermonecrosis *in vivo*. Treatment of *S. aureus* skin infections involves incision and drainage, followed by antibiotic use (David and Daum, 2017). NaP might be applied to the abscess after incision and drainage to control *S. aureus* growth. Moreover, as combination therapy is predicted to be less prone to resistance (Zimmermann et al., 2007), NaP might be used in combination with antibiotics that target D-alanine motifs or other motifs of the bacterial cell wall. In conclusion, we suggest an alternative strategy using propionate, together with a D-alanylation inhibitor, to control antibiotic-resistant *S. aureus* infections.

## Data Availability

The raw data supporting the conclusions of this manuscript will be made available by the authors, without undue reservation, to any qualified researcher.

## Ethics Statement

This study was carried out in accordance with the recommendations of Institutional Biosafety Committee of Seoul National University. Animal experiments were conducted under the approval of Institutional Animal Care and Use Committee of Seoul National University (SNU-170518-5 and SNU-181002-2).

## Author Contributions

SJ, HK, and SH designed the research. SJ, HK, and AK performed the experiments. SJ, HK, and SH analyzed and interpreted the data. SJ, HK, C-HY, and SH wrote and reviewed the manuscript.

## Conflict of Interest Statement

The authors declare that the research was conducted in the absence of any commercial or financial relationships that could be construed as a potential conflict of interest.
