# Supplementary material for: Propionate Ameliorates Staphylococcus aureus Skin Infection by Attenuating Bacterial Growth
Source: Front Microbiol. 2019 Jun 18;10:1363. doi: 10.3389/fmicb.2019.01363 (PMC6591440; doi:10.3389/fmicb.2019.01363)
Supplement: Supplementary file 1 [file Presentation_1.PPTX]

## Slide 1
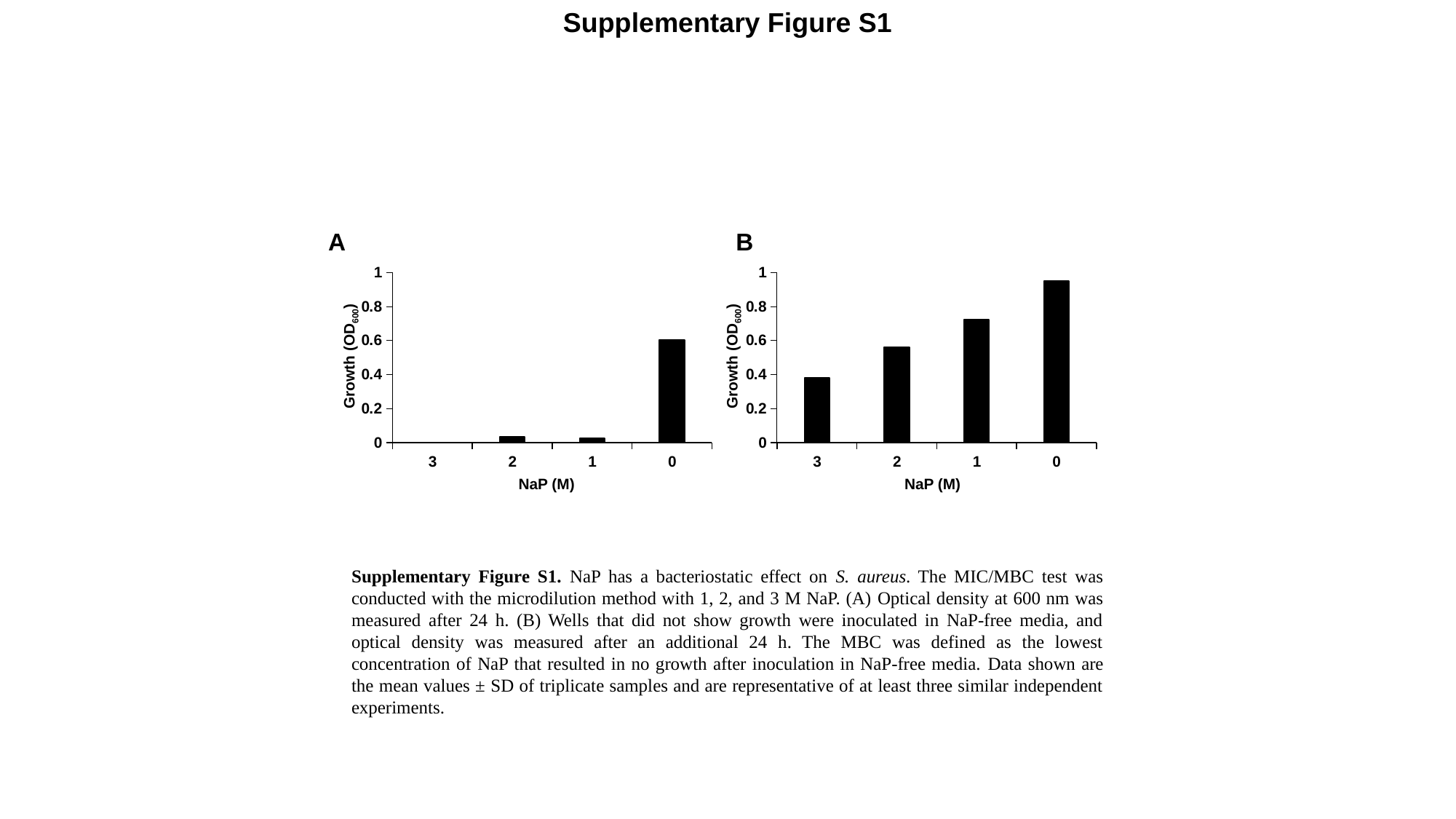

Supplementary Figure S1
A
B
### Chart
| Category | |
|---|---|
| 3 | 0.0014333333333333305 |
| 2 | 0.03366666666666667 |
| 1 | 0.024500000000000004 |
| 0 | 0.6025333333333334 |
### Chart
| Category | |
|---|---|
| 3 | 0.3815 |
| 2 | 0.5622999999999999 |
| 1 | 0.7227666666666667 |
| 0 | 0.9507 |Growth (OD600)
Growth (OD600)
NaP (M)
NaP (M)
Supplementary Figure S1. NaP has a bacteriostatic effect on S. aureus. The MIC/MBC test was conducted with the microdilution method with 1, 2, and 3 M NaP. (A) Optical density at 600 nm was measured after 24 h. (B) Wells that did not show growth were inoculated in NaP-free media, and optical density was measured after an additional 24 h. The MBC was defined as the lowest concentration of NaP that resulted in no growth after inoculation in NaP-free media. Data shown are the mean values ± SD of triplicate samples and are representative of at least three similar independent experiments.

## Slide 2
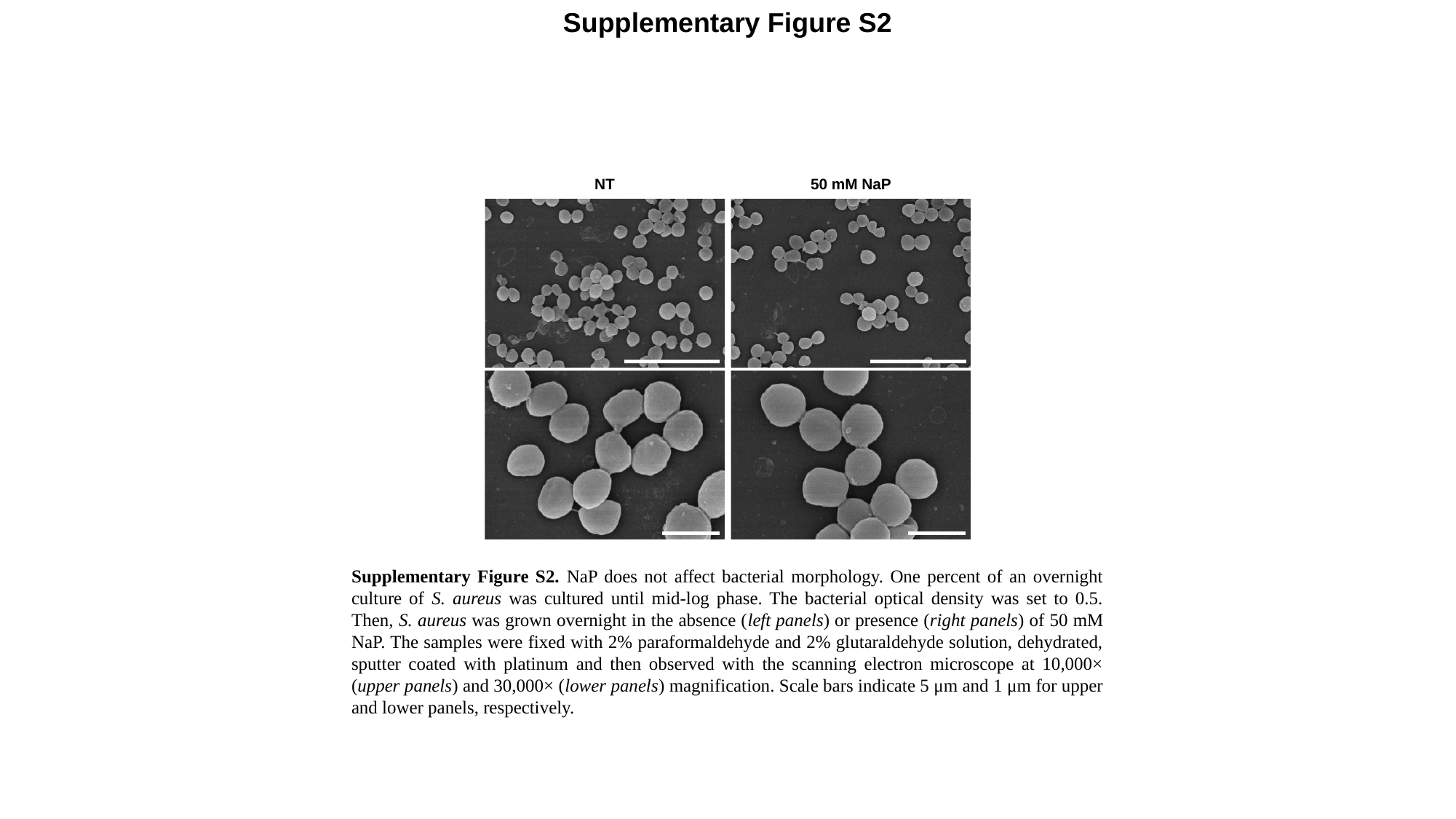

Supplementary Figure S2
NT
50 mM NaP
Supplementary Figure S2. NaP does not affect bacterial morphology. One percent of an overnight culture of S. aureus was cultured until mid-log phase. The bacterial optical density was set to 0.5. Then, S. aureus was grown overnight in the absence (left panels) or presence (right panels) of 50 mM NaP. The samples were fixed with 2% paraformaldehyde and 2% glutaraldehyde solution, dehydrated, sputter coated with platinum and then observed with the scanning electron microscope at 10,000× (upper panels) and 30,000× (lower panels) magnification. Scale bars indicate 5 μm and 1 μm for upper and lower panels, respectively.

## Slide 3
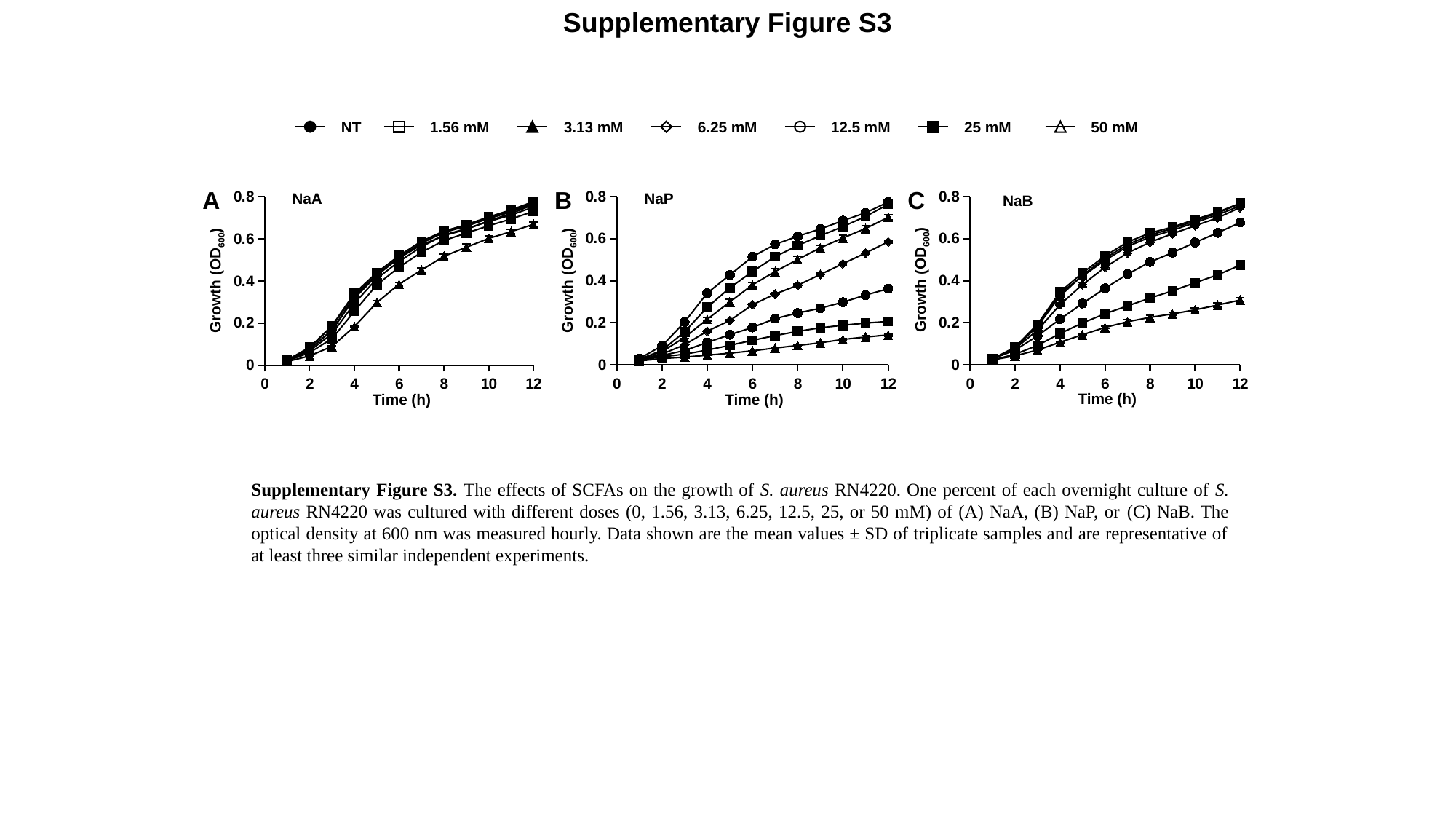

Supplementary Figure S3
NT
1.56 mM
3.13 mM
6.25 mM
12.5 mM
25 mM
50 mM
### Chart
| Category | NT | 1.56 mM | 3.125 mM | 6.25 mM | 12.5 mM | 25 mM | 50 mM |
|---|---|---|---|---|---|---|---|B
NaP
Growth (OD600)
Time (h)
### Chart
| Category | NT | 1.56 mM | 3.125 mM | 6.25 mM | 12.5 mM | 25 mM | 50 mM |
|---|---|---|---|---|---|---|---|C
NaB
Growth (OD600)
Time (h)
### Chart
| Category | NT | 1.56 mM | 3.125 mM | 6.25 mM | 12.5 mM | 25 mM | 50 mM |
|---|---|---|---|---|---|---|---|A
NaA
Growth (OD600)
Time (h)
Supplementary Figure S3. The effects of SCFAs on the growth of S. aureus RN4220. One percent of each overnight culture of S. aureus RN4220 was cultured with different doses (0, 1.56, 3.13, 6.25, 12.5, 25, or 50 mM) of (A) NaA, (B) NaP, or (C) NaB. The optical density at 600 nm was measured hourly. Data shown are the mean values ± SD of triplicate samples and are representative of at least three similar independent experiments.

## Slide 4
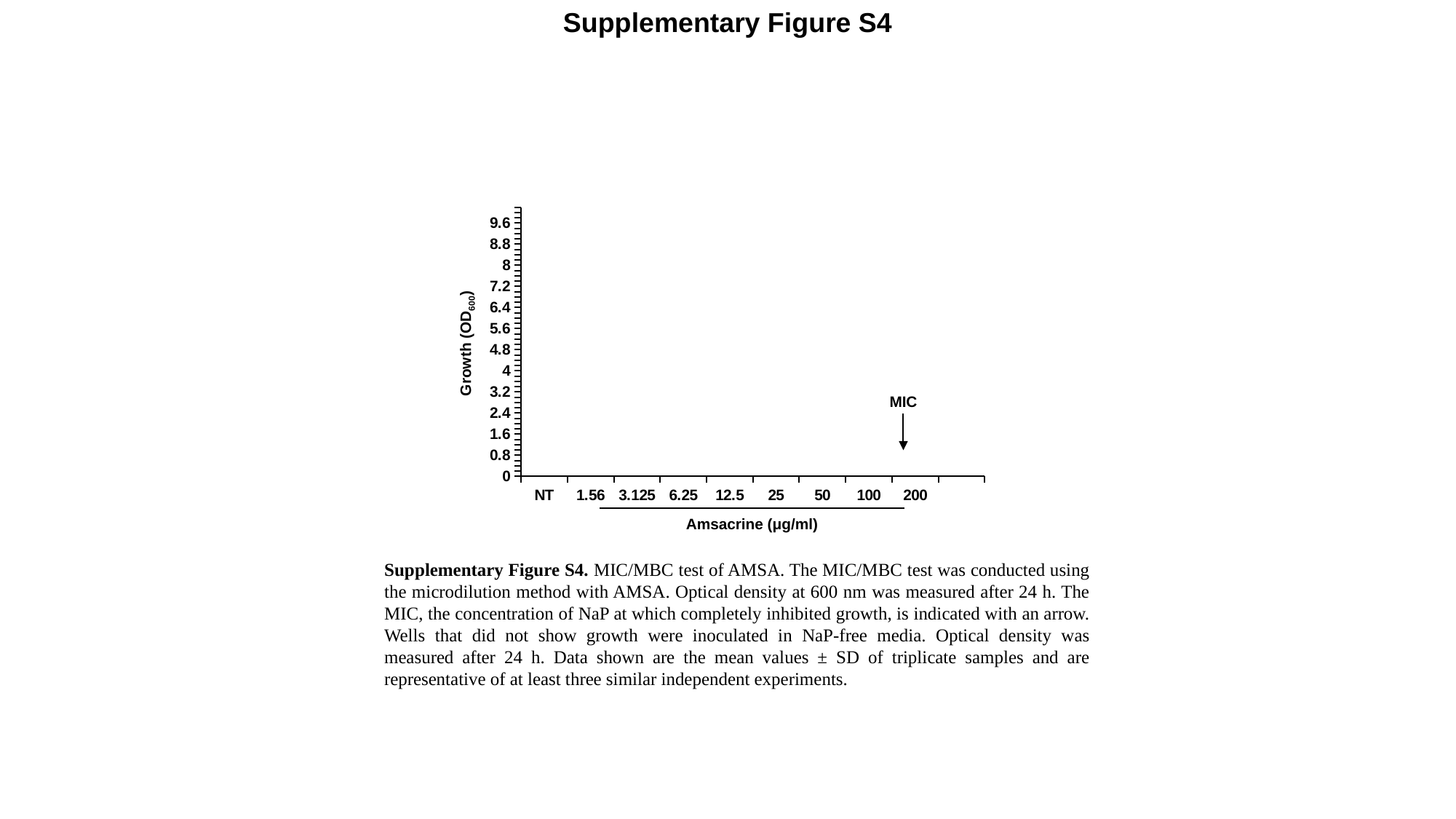

Supplementary Figure S4
### Chart
| Category | AVG |
|---|---|
| NT | 0.8902000000000001 |
| 1.56 | 0.644 |
| 3.125 | 0.5954333333333333 |
| 6.25 | 0.6005 |
| 12.5 | 0.5426 |
| 25 | 0.5416666666666666 |
| 50 | 0.44206666666666666 |
| 100 | -0.007300000000000001 |
| 200 | 0.001999999999999997 |Growth (OD600)
MIC
Amsacrine (μg/ml)
Supplementary Figure S4. MIC/MBC test of AMSA. The MIC/MBC test was conducted using the microdilution method with AMSA. Optical density at 600 nm was measured after 24 h. The MIC, the concentration of NaP at which completely inhibited growth, is indicated with an arrow. Wells that did not show growth were inoculated in NaP-free media. Optical density was measured after 24 h. Data shown are the mean values ± SD of triplicate samples and are representative of at least three similar independent experiments.

## Slide 5
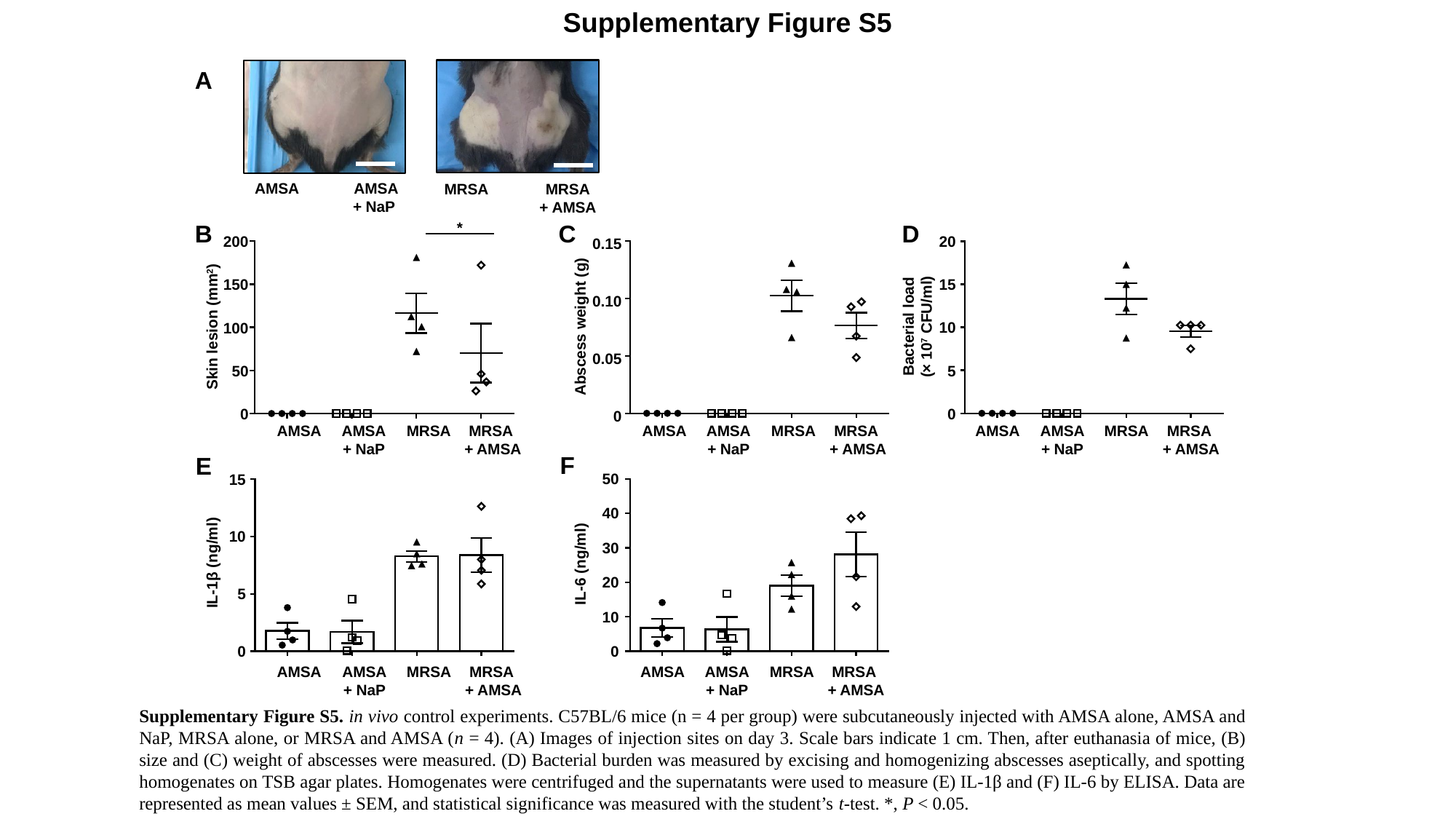

Supplementary Figure S5
A
AMSA
 AMSA
+ NaP
MRSA
+ AMSA
MRSA
*
C
D
B
200
20
0.15
15
150
0.10
Bacterial load
(× 107 CFU/ml)
10
100
Abscess weight (g)
Skin lesion (mm2)
0.05
5
50
0
0
0
AMSA
AMSA
+ NaP
MRSA
MRSA
+ AMSA
AMSA
AMSA
+ NaP
MRSA
MRSA
+ AMSA
AMSA
AMSA
+ NaP
MRSA
MRSA
+ AMSA
F
E
50
15
40
10
30
IL-1β (ng/ml)
IL-6 (ng/ml)
20
5
10
0
0
AMSA
AMSA
+ NaP
MRSA
MRSA
+ AMSA
AMSA
AMSA
+ NaP
MRSA
MRSA
+ AMSA
Supplementary Figure S5. in vivo control experiments. C57BL/6 mice (n = 4 per group) were subcutaneously injected with AMSA alone, AMSA and NaP, MRSA alone, or MRSA and AMSA (n = 4). (A) Images of injection sites on day 3. Scale bars indicate 1 cm. Then, after euthanasia of mice, (B) size and (C) weight of abscesses were measured. (D) Bacterial burden was measured by excising and homogenizing abscesses aseptically, and spotting homogenates on TSB agar plates. Homogenates were centrifuged and the supernatants were used to measure (E) IL-1β and (F) IL-6 by ELISA. Data are represented as mean values ± SEM, and statistical significance was measured with the student’s t-test. *, P < 0.05.
